# Monitoring the Fate of Orally Administered PLGA Nanoformulation for Local Delivery of Therapeutic Drugs

**DOI:** 10.3390/pharmaceutics11120658

**Published:** 2019-12-06

**Authors:** Lucia Morelli, Sara Gimondi, Marta Sevieri, Lucia Salvioni, Maria Guizzetti, Barbara Colzani, Luca Palugan, Anastasia Foppoli, Laura Talamini, Lavinia Morosi, Massimo Zucchetti, Martina Bruna Violatto, Luca Russo, Mario Salmona, Davide Prosperi, Miriam Colombo, Paolo Bigini

**Affiliations:** 1 NanoBioLab, Dipartimento di Biotecnologie e Bioscienze, Università di Milano-Bicocca, Piazza della Scienza 2, 20126 Milano, Italy; luciaagnese.morelli@libero.it (L.M.); lucia.salvioni@unimib.it (L.S.); maria.guizzetti@gmail.com (M.G.); colzanib@gmail.com (B.C.); davide.prosperi@unimib.it (D.P.); 2 IRCCS Mario Negri Institute for Pharmacological Research; Via Mario Negri 2, 20157 Milano, Italy; sara.gimondi@gmail.com (S.G.); m.sevieri@campus.unimib.it (M.S.); laura.talamini@marionegri.it (L.T.); lavinia.morosi@marionegri.it (L.M.); massimo.zucchetti@marionegri.it (M.Z.); martina.violatto@marionegri.it (M.B.V.); luca.russo@marionegri.it (L.R.); mario.salmona@marionegri.it (M.S.); 3 Dipartimento di Scienze Farmaceutiche, Università di Milano, Via Giuseppe Colombo 71, 20133 Milano, Italy; luca.palugan@unimi.it (L.P.); anastasia.foppoli@unimi.it (A.F.); 4 Nanomedicine Laboratory, ICS Maugeri S.p.A. SB, Via S. Maugeri 10, 27100 Pavia, Italy

**Keywords:** PLGA-NPs, nanomedicine, gastrointestinal tract, paclitaxel, in vivo imaging

## Abstract

One of the goals of the pharmaceutical sciences is the amelioration of targeted drug delivery. In this context, nanocarrier-dependent transportation represents an ideal method for confronting a broad range of human disorders. In this study, we investigated the possibility of improving the selective release of the anti-cancer drug paclitaxel (PTX) in the gastro-intestinal tract by encapsulating it into the biodegradable nanoparticles made by FDA-approved poly(lactic-*co*-glycolic acid) (PLGA) and coated with polyethylene glycol to improve their stability (PLGA-PEG-NPs). Our study was performed by combining the synthesis and characterization of the nanodrug with in vivo studies of pharmacokinetics after oral administration in mice. Moreover, fluorescent PLGA-nanoparticles (NPs), were tested both in vitro and in vivo to observe their fate and biodistribution. Our study demonstrated that PLGA-NPs: (1) are stable in the gastric tract; (2) can easily penetrate inside carcinoma colon 2 (CaCo_2_) cells; (3) reduce the PTX absorption from the gastrointestinal tract, further limiting systemic exposure; (4) enable PTX local targeting. At present, the oral administration of biodegradable nanocarriers is limited because of stomach degradation and the sink effect played by the duodenum. Our findings, however, exhibit promising evidence towards our overcoming these limitations for a more specific and safer strategy against gastrointestinal disorders.

## 1. Introduction

The generation of therapeutic methods that can improve the organ specificity of many different types of therapeutic agents is one of the major challenges of 21st-century pharmacology [1]. The availability of transport systems that can successfully improve the targeted release of administered substances would be, indeed, crucial in mitigating or even virtually eliminating the toxic side effects of already existing therapeutics. These improved transport systems can be used to limit the off-target spread and, consequently, reduce the potential side effects deriving from an unnecessary tropism [2]. Several strategies have been employed in the last years to improve the accuracy of drug targeting. Quite recently, our group and others have demonstrated that the systemic administration of anti-inflammatory and immunomodulatory agents, such as corticosteroids, can have a safer and more effective pharmacological profile when linked to a nanometric vector [3,4]. The potential prospects of nanocarriers for pharmacological purposes is extremely versatile complex. It is widely known that even the slightest modification of basic physicochemical strictures, such as the dimension, shape, and surface of the material itself, can indeed lead to a myriad of different results—causing a very vast range of reactions amongst the cells and organs of the host [5]. One of the primary and certainly most prominent, therapeutic strategies is the incorporation of the anti-neoplastic drug doxorubicin into liposomes for the treatment of tumors [6]. The first generation of nanoparticles (NPs) used for these purposes are mainly liposomes, polymeric micelles, and conjugates.

Since the turn of the century, the encapsulation of anti-neoplastic cytotoxic molecules inside NPs has been occurring with greater frequency. In 2004, an original formulation for the creation of nanocarriers to transport the anti-cancer drug paclitaxel (PTX) was developed [7,8]. In that case, different from other carriers, the biodegradable copolymer poly(lactic-*co*-glycolic acid) (PLGA) was utilized. In a more recent study, furthermore, it was shown that the systemic administration of PTX inside poly(lactic-*co*-glycolic acid)-polyethylene glycol-nanoparticles (PLGA-PEG-NPs), when functionalized with ANTI-EGFR, dramatically slows disease progression in triple-negative breast cancer-bearing mice, thus confirming the high efficacy, safety, and tenability of this material [9]. 

Nanoparticles utilizing FDA-approved PLGA, exhibit some important advantages. In particular, their biocompatibility causes low systemic toxicity in comparison to other polymers [10] and is highly versatile, allowing them to encapsulate various drugs, including small molecules and therapeutic biologics [11,12,13]. The importance of PLGA nanocarriers in biomedicine is further supported by evidence of its potential benefits in assisting drug transportation across biological barriers [14,15]. Most of their applications concern intravenous administration [16], but, in the last decade, many studies have been published regarding their potential efficacy in oral administration of drugs, even if the absorption efficiency needs to be more deeply investigated and optimized [17].

Although the majority of studies dealing with biodistribution, disease targeting, and therapeutic efficacy of NPs-based drug delivery systems has been conducted using systemic administration of nanoformulation, more recently the exploration of alternative routes, including topical [18,19], intranasal [20], intratracheal [21], and oral administration [22], are gaining increasing recognition.

Building on these advances, this work aimed to characterize the biodistribution of PTX alone or encapsulated in PEGylated PLGA-NPs after oral administration in healthy mice. This approach stems from the hope that, in so doing, the passage of the drug from the gastrointestinal (GI) tract to the bloodstream might be reduced, although not completely eliminating its release in the gut. While the active gastroduodenal absorption of PTX on oral ingestion is quite low, it is not possible to totally exclude systemic toxicity of both circulating cells and filter organs in the case of high doses or repeated cycles of chemotherapy. PLGA-NPs could, therefore, be useful to both protect the drug against gastric degradation and, at the same time, reduce the uptake by mucosae villi or Peyer’s patches and the consequent release into the blood, while PEG grafting is expected to improve both the NPs stability and mucus-permeating properties in the GI tract [23,24]. In this study, PLGA-PEG-NPs were orally administered either loaded with PTX or labeled with a fluorophore (Rhodamine B), which allowed for the longitudinal tracking of NPs in both gastrointestinal and peripheral organs. This study could be of interest to understand the potentials of local delivery of anti-cancer agents in GI tumors by the oral administration of NPs, minimizing nonspecific absorption.

## 2. Materials and Methods

### 2.1. Nanoparticles Synthesis

#### 2.1.1. Materials

PLGA (Resomer^®^ RG 504 H, 50:50 lactide:glycolide, acid terminal, MW 38,000–54,000 g/mol), paclitaxel (PTX, MW 853.91 g/mol), rhodamine B (RhB, MW 479.01 g/mol); polyvinlyl alcohol (PVA, MW 9000–10,000 g/mol, 80% hydrolyzed); *N,N*′-dicyclohexylcarbodiimide (DCC, MW 206.33 g/mol, 99%); *N*-hydroxysuccinimide (NHS, MW 115.09 g/mol, 98%); dimethylamino pyridine (DMAP, MW 122.17 g/mol, 99%); ninhydrin (MW 178.14 g/mol); ethanolamine (MW 61.08 g/mol, ≥98%); dichloromethane (DCM, ≥99.8%); dimethyl sulfoxide (DMSO, ≥99.9%); acetonitrile anhydrous (ACN, 99.8%); methanol (MeOH, ≥99.9%) were purchased from Sigma–Aldrich Co (St Louis, MO, USA). Chloroform (≥99.8%) was purchased from Honeywell Riedel-de-Haën™ (Charlotte, NC, USA)). Poly (ethylene glycol) diamine (NH_2_-PEG-NH_2_, 6000 Da) was obtained from Rapp Polymer GmbH, (Tuebingen, Germany).

#### 2.1.2. PLGA-PEG-RhB Synthesis

For activation of the carboxylic groups of PLGA, the polymer was dissolved in DCM (15 mL, 40 mg/mL) and the reaction was performed in a round flask for 4 h (250 rpm, RT, under inert gas) after the addition of DCC (30 mg) and NHS (15 mg). Then, the solution was diluted with diethyl ether, and the polymer was collected after precipitation (20 min, 6200× *g*, 4 °C) and evaporation of the residual organic phase. Next, the conjugation with NH_2_-PEG-NH_2_ (molar ratio PEG/PLGA = 2.7) was performed in 10 mL of CHCl_3_ overnight (250 rpm, RT, under inert gas). To get rid of the unconjugated PEG, methanol (30 mL) was added, and the polymer was collected after precipitation twice [25]. The residual organic phase was then evaporated under reduced pressure, and the weight yield calculated (68.99 ± 14.94%). The degree of labeling was determined using the colorimetric ninhydrin assay (primary amine detection): Notably, 15 mg of the product (PLGA-PEG) was solubilized in DMSO (400 µL) then diluted with 100 µL of ninhydrin solution (3.5 mg/mL). Ethanolamine (18.75 mg/mL) and PLGA solutions were used as positive and negative controls, respectively. The samples were then incubated for 1 h at 65 °C (400 rpm), and the colored conjugate was detected by UV-Vis spectroscopy (Spectrophotometer Fluormax-Horiba Scientific, Rome, Italy) (λ = 600 nm), and the yield calculated using a calibration curve of ethanolamine standards. The obtained degree of labeling was 71.90 ± 12.70%.

Finally, RhB (molar ratio RhB/PLGA = 6.25) was added to the PLGA-PEG solution in CHCl_3_ (15 mL) with an excess of DCC and DMAP. The reaction was carried out overnight (250 rpm, RT, under inert gas). At the end of the reaction, the solution was evaporated, and the product was solubilized with DCM (10 mL) and purified thrice by precipitation (20 min, 4 °C, 6200× *g*) after diethyl ether addition (20 mL). The reaction yield was 38.16 ± 13.50%. RhB linked was detected by fluorescence spectrometry (λ_exitation_ 555 nm; λ_emission_ 574 nm) and the degree of labeling calculated by the comparison with a calibration curve (1.43 ± 0.46 µg/mg).

#### 2.1.3. Synthesis of PLGA-PEG-RhB-NPs and PTX-PLGA-PEG-RhB-NPs

NPs were synthesized according to the single emulsion method. PLGA-PEG-RhB solution (1 mL, 25 mg/mL in DCM) was emulsified with 8 mL of PVA 2% solution by sonicating twice (Sonifier Sound, Branson Ultrasonics, Shanghai, China; 30 s and 38% intensity) in an ice bath. The product was transferred immediately into a solution of PVA 2% (16 mL), allowing the organic solvent to evaporate (4 h, RT, 750 rpm). For PTX-PLGA-PEG-RhB-NPs synthesis, 5 mg of PTX was dissolved along with the polymer. After curing, NPs were collected by centrifugation at 19,500× *g*, 20 min, 4 °C (Heraeus Fresco 21; Thermo Fisher Scientific, Göteborg, Sweden), washed thrice with double distilled water and freeze-dried through an Alpha 1–2 LD freeze drier (Christ, Memmingen, Germany) at 0.500 mbar, −53 °C, 12 h, without the addition of any cryoprotectant. The process yield was calculated after the freeze-drying process, as the ratio between collected NPs and starting raw materials. 

The amount of PTX loaded in the NPs was determined by HPLC analysis with UV detection (Waters Associates, Milford, MA, USA, model 2487 Variable Wavelength Detector, Wavelength: 230 nm). Briefly, 0.1 mL of the NPs solution was spiked with 5 µg of IS and extracted with 0.5 mL of CH_3_CN. After vortex for 10 s, samples were centrifuged at 13,000 rpm for 10 min. The organic phase was separated and dried under nitrogen, and the residues were dissolved with 250 µL of the mobile phase. Fifty microliters of the reconstituted samples was injected into the HPLC system. The apparatus was equipped with a Symmetry C18 column (5 μm, 4.6 × 150 mm), the mobile phase was composed of 50% ammonium acetate buffer (0.01 M, pH 5), 40% acetonitrile, and 10% methanol with a flux rate of 1.3 mL/min and 30 min run time. NPs solution without drug was used to prepare the calibration curve by the addition of PTX in the range 10–100 µg/mL.

### 2.2. Nanoparticles Characterization

#### 2.2.1. Dynamic Light Scattering (DLS) and Zeta Potential Measurements

All NPs were characterized in terms of size, size distribution, and Zeta potential through dynamic light scattering (DLS, Zetasizer Nano ZS; Malvern Instruments, Cambridge, UK) in ultrapure water at 1 mg/mL, at 25 °C. The scattered light from the NPs in suspension was used to calculate NPs’ hydrodynamic diameter considering medium viscosity. NPs size distribution was described by the polydispersity index (PDI), where PDI ≤ 0.2 corresponds to the monodisperse NPs population.

#### 2.2.2. Scanning Transmission Electron Microscopy (STEM) Analysis

PLGA-PEG-RhB-NPs were observed by a Zeiss SEM-FEG Gemini 500, operating at 30 kV in scanning transmission electron microscopy (STEM) mode (Zeiss, Germany). The NPs suspension was deposited onto a formvar-coated 200-mesh copper grid (Ted Pella, CA, USA), negatively stained with 1% uranyl acetate and allowed to dry before examination.

#### 2.2.3. In Vitro Release Studies

PTX-PLGA-PEG-RhB-NPs equivalent to 8.75 μg of PTX were suspended in 1 mL of Tween/PBS and incubated at 37 °C with constant agitation. At predetermined time points (0.5, 1, 2, 4, 6, and 24 h), the suspension was centrifuged at 19,500× *g* for 15 min to separate NPs pellets and supernatants. PTX in the collected supernatants was analyzed with HPLC equipped with a UV detector (1260 Infinity II Series, Agilent Technologies, Palo Alto, CA, USA) and an Atlantis C18 column (25 cm × 4.6 mm, particle size 5 μm) (Supelco, St. Louis, MO, USA). The mobile phase was a mixture of acetonitrile and water (50:50) run in the isocratic mode at a flow rate of 1 mL/min. PTX was detected at 227 nm. PTX quantitation was performed using a calibration curve in a range of 1.25–40 µg/mL, and the results were expressed as a percentage of cumulative release (mean ± SD; *n* = 4).

#### 2.2.4. Nanoparticles Stability in Different Buffers

The hydrodynamic dimension of the nanocarrier was analyzed in different conditions: Artificial saliva (pH 7.6; KH_2_PO_4_ 1.9 mM, NaHCO_3_ 17 mM, Na_2_HPO_4_ 1.8 mM, NaCl 8.5 mM, KSCN 3.4 mM, Urea 2.2 mM, alpha-amylase 0.007 mM), intestinal fluid (pH 6; KH_2_PO_4_ 49.96 mM, NaOH 0.0154 M, pancreatin 1 g/L), and gastric juice (pH 1.2; NaCl 300 mM, pepsin 0.9 mM, HCl 840 mM) that simulate the media in GI tract. The NPs (15 mg/mL) were incubated for 0, 1, 2, 4, 6, and 24 h and analyzed by DLS. Upon establishing the size stability, the samples were further analyzed with fluorescence spectroscopy (λ_exitation_ 555 nm; λ_emission_ 574 nm) to monitor the stability of RhB conjugation. The release of RhB after 24 h was then calculated after PLGA-PEG-RhB-NPs removal by centrifugation (mean ± SD; *n* = 4).

### 2.3. Cells

Carcinoma colon cells (CaCo_2_) are a cell line derived from human colorectal adenocarcinoma and were purchased from ATCC^®^ HTB-37™. CaCo_2_ cells were grown as a single layer in adhesion in DMEM-High Glucose (Dulbecco′s Modified Eagle Medium High Glucose-Biowest, Nuaillé, France) with the addition of 10% of fetal bovine serum (FBS) and 1% l-glutamine (200 mM), 100 U/mL penicillin, 0.1 mg per mL streptomycin. Cells were maintained at 37 °C and in a 5% CO_2_ humidified atmosphere. The ability of PLGA-PEG-RhB-NPs and free RhB to be internalized by CaCo_2_ cells was evaluated. For each condition, the experiment was conducted in triplicate. Cells were seeded at a density of 40,000 cells/well on 13 mm diameter slides in 24-well plates. Forty-eight hours after sowing, CaCo_2_ cells were incubated with PLGA-PEG-RhB-NPs (100 µg/mL) and with RhB free (0.06 µg/mL) for 1, 4, and 24 h, while the wells destined for control did not receive any treatment. After incubation, the cells were washed with phosphate-buffer saline (PBS) three times and fixed with a 4% paraformaldehyde solution dissolved in PBS (0.1 M, pH 7.4) for 40 min and the vital nuclear dye Hoechst 33258 (2 μg/mL) was added to each well for 45 min. The slides thus obtained were assembled and analyzed with the Olympus BX51 epifluorescence microscope (Olympus, Tokyo, Japan). All acquisition parameters, including laser settings, were kept constant during all scans. To evaluate a possible cytotoxic effect of PLGA-PEG-RhB-NPs and free RhB, the metabolic activity of CaCo_2_ cells was evaluated by RealTime-Glo™ MT Cell Viability Assay (Promega, Madison, WI, USA). Cells were seeded at 16,000 cells/well in 96 opaque-walled tissue culture plates and maintained at 37 °C. The RealTime-Glo reagents were added at the same time as the test compounds, according to the manufacturer’s protocol. At selected time points (4 and 24 h), the cell viability was monitored by a plate-reading luminometer (GloMax^®^ Discover Microplate Reader, Promega, Madison, WI, USA). For each condition, 6 replicates were prepared. The viability was expressed as a percentage compared to non-treated cells.

### 2.4. Animals

All procedures involving animals and their health were conducted so as to minimize the number of mice used and their collateral suffering, in accordance with institutional guidelines, national laws (DL n. 24, 4 March 2014; Authorization No. 19/2008 A) and international laws and agreements (EEC Council Directive 2010/63, 6 August 2013; NIH Guide to the Care and Use of Laboratory Animals, US National Research Council, 2008). The research project was first reviewed by the Internal Ethics Committee of the Mario Negri Institute and was subsequently approved by the ministry and designated with the before mentioned code (42/2016-PR).

All animals were housed in SPF (specific pathogen-free) conditions. The housing rooms had a temperature of 22 ± 1 °C, relative humidity values ± of 50 ± 10% and a 12 h light/dark cycle. Furthermore, the animals were kept in cages with free access to water and food.

### 2.5. Treatments

As regards pharmacokinetics, six weeks old CD1 mice were treated by oral gavage with 20 mg/kg of Cremophor PTX (*n* = 16) or PTX-PLGA-PEG-RhB-NPs (*n* = 16), three animals were treated with saline solution as control. Blood was collected in heparinized tubes, 30 min, 1, 4, and 24 h after treatment (4 animals for each group) and centrifuged to obtain plasma. After the mice were sacrificed, the stomach, duodenum, colon, and liver were collected and stored at −20 °C until analysis.

For the biodistribution studies, we recruited 5 animals for each experimental group. To reduce background fluorescence due to the food, mice were fed an AIN-76A diet without alfalfa (Mucedola s.r.l., Settimo Milanese, Italy) for two weeks before the analyzes. The dose of the different formulations was standardized on the quantity of RhB present and fixed at 0.6 mg/kg mouse based on previous experimental studies. Vehicle treated mice received the same volume of saline solution. Mice were sacrificed at 1, 4, or 24 h to follow the fate of RhB. The sacrifice was performed by cervical dislocation, and the stomach, intestine, liver, and blood were collected to perform ex vivo analyses. Plasma for fluorometric analysis was obtained from the blood collected. Finally, a piece of liver and GI tracts of three animals for each experimental group were frozen at −80 °C for cryostatic sections and histological analysis.

### 2.6. Molecular Imaging and Histology

The in vivo biodistribution of the different formulations was monitored over time using an optical fluorescence imaging system (IVIS Lumina XRMS, PerkinElmer, Waltham, MA, USA). Ex vivo scans of organs from mice sacrificed at 1, 4, and 24 h after the treatment were performed by the same instrument. The following acquisition parameters were used: Excitation filter: 580 nm, emission filter: 620 nm, exposure time: Auto, binning factor: Medium, f/Stop: 2, Field of View: D (for the gastro line—intestinal), C (for peripheral organs). Very importantly, the Living Image Software 4.3.1 (Perkin Elmer, Waltham, MA, USA) conjugated with the spectral unmixing system was used to separate the RhB signal from tissue autofluorescence, image processing, and fluorescence signal quantification analysis.

Longitudinal sections of 20 μm of thickness were prepared and then, after adhesion in glass slides, were incubated with a PBS solution of Hoechst 33258 (2 µg/mL, Sigma–Aldrich) for 45 min and, after three washes in PBS, observed at the Microscopy Virtual Slide (Olympus, Tokyo, Japan), to obtain rapid organ scans of the whole section with high anatomical resolution.

### 2.7. NPs Characterization from Homogenates and Biological Fluids

The fluorescence of PLGA-PEG-RhB-NPs was analyzed in tissues explanted from previously treated animals. The analyzed tissues were liver, stomach, intestine, and plasma. Each tissue sample was weighed, homogenized in PBS 1X according to a 1:4 weight ratio, and centrifuged at 1200 rpm for 10 min at 4 °C. For all samples, the analysis was performed using the Infinite^®^ M200 multimode plate reader exciting at the wavelength of 500 nm and recording the signal at a range of emission wavelength from 550 to 560 nm. For the stability studies in solutions mimicking gastrointestinal fluids, the NPs were incubated in stock solutions as previously described [26].

### 2.8. Pharmacokinetics

The total concentration of PTX in the different biological matrices was determined by HPLC-UV, as previously described [27]. For the determination of PTX in organs, tissues were previously homogenized in 0.2 M CH_3_COONH_4_ pH 4.5. Each study sample (0.3 mL for plasma and 0.5 mL for homogenate tissues) was assayed together with five points of a standard calibration curve prepared in the corresponding control biological matrix obtained from untreated mice at concentrations ranging from 0.05 to 5 µg/sample. The limits of quantification (LOQ) were 0.16 µg/mL and 0.6 µg/g for plasma and organs, respectively.

### 2.9. Statistical Analysis

All data were expressed as mean ± SD, Student’s *t*-test and *p* values were done using the GraphPad Prism version 6.00 for Windows (Graph-Pad Software, San Diego, CA, USA).

## 3. Results and Discussion

### 3.1. NPs Synthesis and Characterization

The protocol for the polymer modification was set up, and PLGA-PEG-RhB was employed to synthesize the NPs to be used as carriers for PTX. The covalently bound RhB chromophore allowed the tracking of the PLGA fate in vivo. The single emulsion method allowed us to obtain both unloaded and PTX-loaded NPs, having controlled size and homogenous size distributions (Table 1). As measured by DLS analysis, the encapsulation of PTX did not significantly change the dimensional features of NPs. Moreover, a negative Z-potential was observed as opposed to PLGA-PEG-NPs, where the free amines of PEG determined the surface properties. However, a small difference in net negative charge values between PLGA-PEG-RhB-NPs and PTX-PLGA-PEG-RhB-NPs was attributable to the intramolecular reorganization of portions of the polymer chains due to hydrophobic interaction with PTX. PTX loaded NPs were further characterized by STEM, which showed pseudospherical shaped nanoparticles with the size around 200 nm (Figure S1). PTX encapsulation efficiency detected by HPLC analysis was 10.87 ± 1.13%, while the calculated loading efficiency was 4.77 ± 0.15%, in line with previous studies [28]. The drug release performance was evaluated, and the test was conducted in PBS containing 0.2 *v*/*v* % Tween 80, considering both its poor solubility and the analysis detection limits [29]. As previously reported with similar experimental settings, a fast PTX dissolution was observed (80% within 4 h) (Figure S2) [30].

#### Stability in Mimicking Biological Fluids

**Figure 1:**
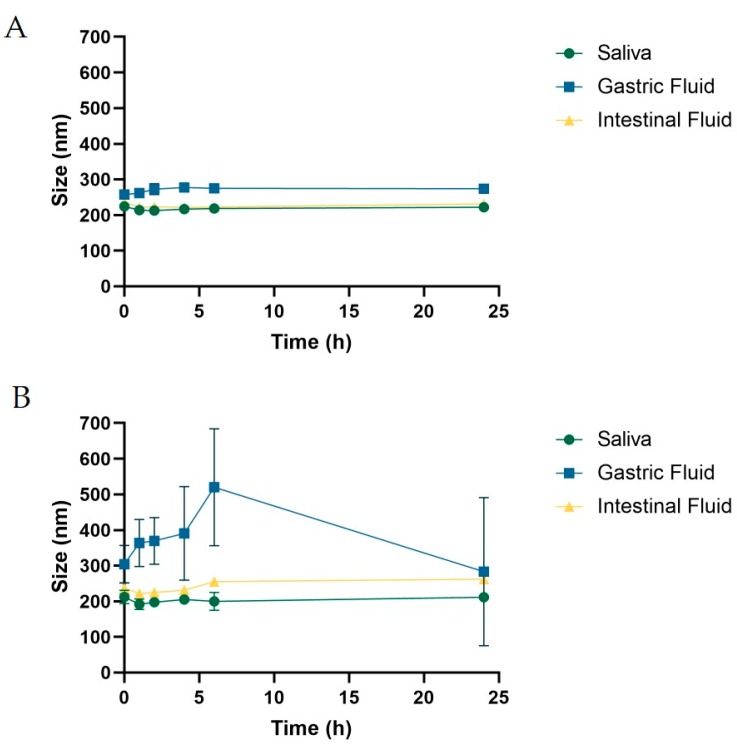
Graphics showing the diameter, measured by dynamic light scattering (DLS), after incubation of (**A**) pegylated or (**B**) not pegylated poly(lactic-*co*-glycolic acid)-nanoparticles (PLGA-NPs) in artificial fluids mimicking the saliva (green lines), the gastric juice (blue lines), or proximal intestinal fluid (yellow line). For all conditions, the measurements were carried out at 1, 2, 4, 6, 24 h from the starting point.

The first study was carried out to verify the potential role of PEG on the fate of PLGA-NPs in solutions mimicking saliva, gastric, and proximal intestinal fluid, respectively (Figure 1A,B). Longitudinal measurement of the NPs size by DLS was possible because, diversely from the serum, they are very poor in macromolecules, and the interference on the recording is almost zero. Figure 1A shows that the presence of the PEG confers to PLGA-NPs a long-lasting stability for at least 24 h after incubation. In contrast, the lack of PEG rapidly induced aggregation in NPs incubated in gastric fluid and, to a lesser extent in intestinal fluid. Since we aimed to conserve considerable stability up until the large intestine, we decided to carry on our studies using pegylated PLGA-NPs exclusively.

**Figure 2:**
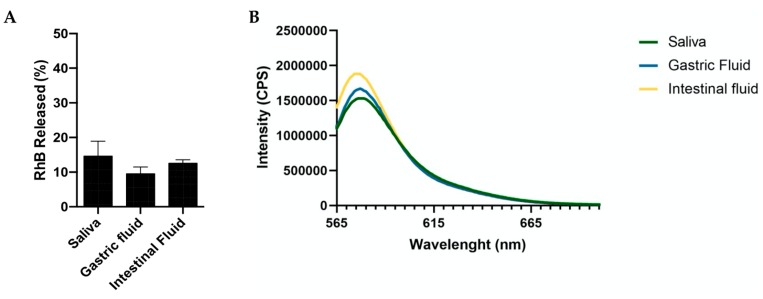
Graphics showing (**A**) the amount of RhB released from PLGA-PEG-RhB-NPs after 24 h incubation in artificial fluids mimicking the saliva, the gastric juice, or proximal intestinal fluid (mean ± SD; *n* = 5); (**B**) emission spectra of poly(lactic-*co*-glycolic acid)-polyethylene glycol-RhB-nanoparticles (PLGA-PEG-RhB-NPs) incubated in different media.

Then, the stability of the RhB conjugation was performed by measuring the dye release in the same conditions (Figure 2A). After 24 h of incubation, the released RhB was below 15% in all cases, suggesting the system reliability for in vitro and in vivo NPs tracking. Additionally, as reported in Figure 2B, the fluorescence intensity of PLGA-PEG-RhB-NPs was not greatly affected by the incubation media showing that, once conjugated, the dye abolishes its pH-dependent emission properties [31].

### 3.2. Pharmacokinetics

**Figure 3:**
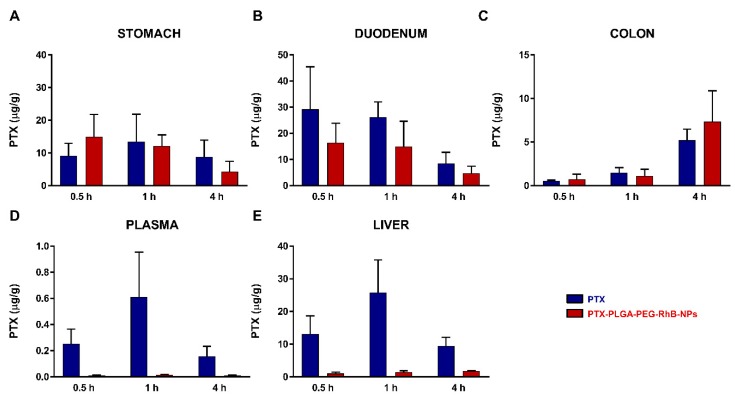
Comparison of the paclitaxel (PTX) distribution in (**A**) stomach, (**B**) duodenum, (**C**) colon, (**D**) plasma, and (**E**) liver of mice after administration of a single treatment of PTX or PTX-PLGA-PEG-RhB-NPs (20 mg/kg p.o.). The bars are the mean value ± SD (*n* = 4).

To understand the possible influence of the nanoformulate on the biodistribution of drugs, we performed a pharmacokinetic study of the well-known anti-cancer agent, paclitaxel, administered orally as a free drug (PTX) or loaded into NPs (PTX-PLGA-PEG-RhB-NPs). PTX release Figure 3 shows the levels of PTX measured in the stomach (A), in the duodenum (B) and in the colon (C) of mice sacrificed 30 min, 1 and 4 h after treatment with PTX free (blue bars) or with PTX-PLGA-PEG-RhB-NPs (red bars). The drug concentration in the samples collected at 24 h after treatment resulted under the limit of detection. Overall, the drug concentration measured in all the gastrointestinal tissues was comparable between the two formulations and higher in the stomach and duodenum compared to the colon. Despite the comparable tissue drug level, interestingly, the nano-formulation reduced the systemic absorption of the drug. The measurement of the plasmatic levels, in fact, showed a clear reduction of systemic absorption of PTX when administered as PTX-PLGA-PEG-RhB-NPs, leading to a consequent reduction of liver accumulation, as shown in Figure 3D,E. Similarly to previous results achieved by our group [32,33], no evidence of acute toxicity was observed in mice receiving the single administration of PTX-PLGA-PEG-RhB-NPs.

### 3.3. NPs Biodistribution and Nanosafety

To better understand the mechanisms that govern the different behavior of the free and NPs-encapsulated drug, we decided to monitor the NPs transit at the digestive tract level, the interactions with the gastric and intestinal structures, the possible transition to the bloodstream and penetration into filter organs by marking PLGA with a fluorescent molecule, RhB. Since the monitoring of biodegradable NPs through an indirect method, such as fluorescence analysis, presents the risk of artifacts caused by the dye release or degradation [34], all studies were carried out by comparing animals that received PLGA-PEG-RhB-NPs with those animals treated with the same dose of free RhB. As the in vitro studies suggested, the stability and reliability of this indirect approach (see Figure 2), enable us to obtain further evidence from the biodistribution study that explains the different behavior between an orally administered small molecule and our nanocarrier.

**Figure 4:**
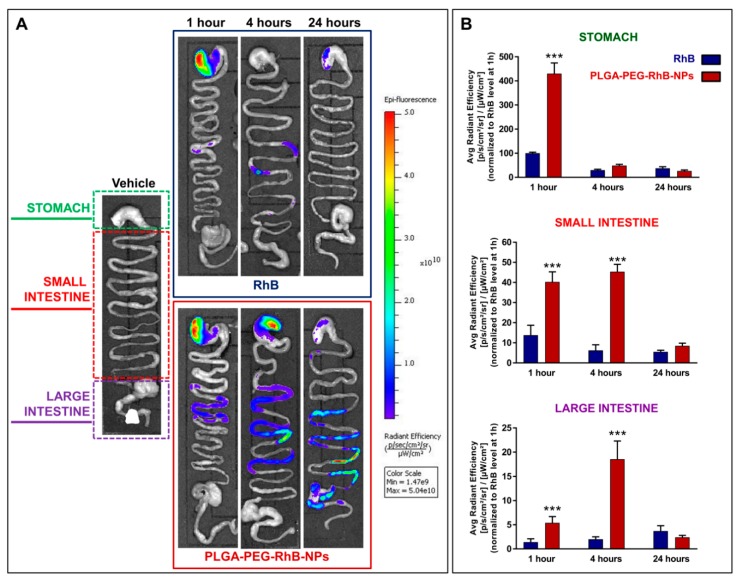
Signal distribution in the gastrointestinal (GI) tract of mice treated with RhB or PLGA-PEG-RhB-NPs. (**A**) Representative ex vivo scanning of the excised organs after washing with saline solutions to remove debris of feces. Animals were sacrificed at 1, 4, and 24 h after the treatment with the same dose of RhB. In the left column, a vehicle-treated mouse was shown to demonstrate the lack of the auto fluorescent component in this analysis. The interval of fluorescence signal intensity associated with the scale of colors is reported on the right. Five animals for each experimental group were used. (**B**) The quantification of the signal associated with the treatment was performed, dividing each sample into three tracts, as shown in the panel. The bars are the value of signal normalized to 100, considering the mean value measured in the stomach of mice treated with RhB alone and sacrificed 1 h after the oral administration. The bars are the mean value ± SD (*n* = 5). The Student’s *t*-test was used to compare the levels between the two groups for each time point. *** *p* < 0.0001.

Figure 4A shows the distribution of the signal associated with the GI tract in animals sacrificed 1, 4, and 24 h after treatment with the vehicle (left) or RhB (upper blue panel), and PLGA-PEG-RhB-NPs (lower red panel), respectively. The power of the excitation laser was set in vehicle-treated mice to avoid any possible overlapping between RhB and tissue autofluorescence. In each panel, it is possible to see an upper region shaped like a sack, which represents the stomach. The small intestine is the snake-like shape in the middle. The enlargement in the last part of the intestine includes cecum, colon, and rectum and is called “large intestine”. The signal associated with RhB is clearly detectable in both groups receiving the same amount of dye. As expected, in both groups, it is also possible to see a progressive decay of signal and a shift from the distal part of the GI tract. However, in mice treated with NPs, the presence of the dye was more persistent, in particular in the distal part of the small intestine and in the large intestine. The quantification of the signal (Figure 4B) confirmed this observational study: In particular, the levels of the signal in the intestine at the 4th hour after the treatment was markedly higher in animals receiving the nanoformulation.

To better understand the interaction between PLGA-PEG-RhB-NPs and the GI tract structures, histological evaluation was carried out, also exploiting the presence of RhB to track them along the anatomic path and their development in time. Even in this case, the same doses of RhB free were injected into a further group of mice to exclude any possible misinterpretation of the results due to the possible loss of NPs stability and the release of the dye.

**Figure 5:**
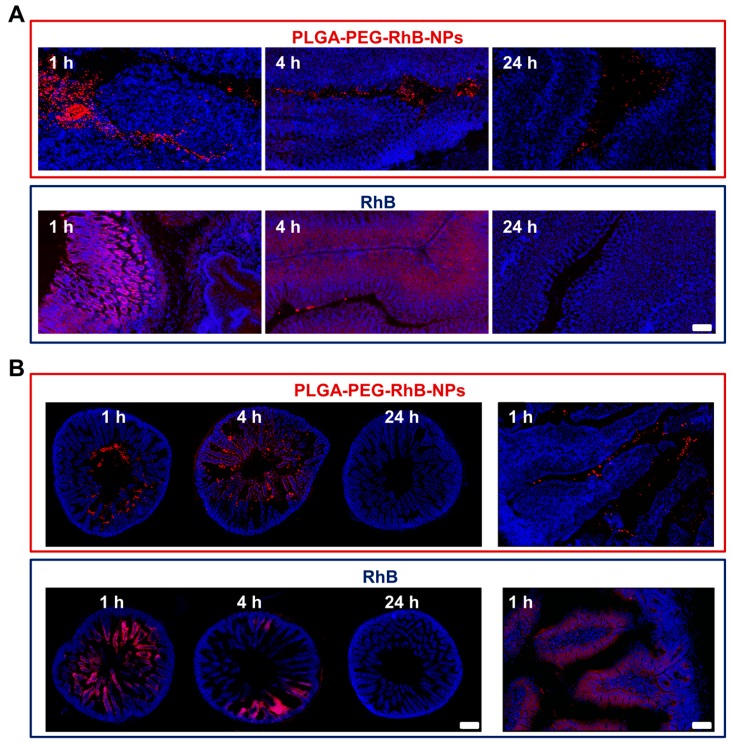
Representative images showing the distribution of RhB in (**A**) stomach and (**B**) duodenum of mice treated with either PLGA-PEG-RhB-NPs or RhB alone. (**A**) In the upper panels, the localization of signal (red) in gastric sections (blue) is shown 1, 4, and 24 h after the oral administration of PLGA-PEG-RhB-NPs. The same procedure has been used to track the presence of the dye in mice receiving the same amount of RhB (lower panels). Scale bar 100 µm. (**B**) Representative images of the duodenum are shown of PLGA-PEG-RhB-NPsand RhB-treated mice in upper and lower panels, respectively. Scale bar 200 µm. The thicker and more intense blue staining in the periphery of the sections represents the basal layer of the duodenum where the exchanges of tissue/blood occur. The red signal is more concentrated to the center, likely corresponding to the lumen close to the apical side of the villi. A higher magnified picture from a mouse sacrificed 1 h after the ingestion of NPs confirms the weak interaction between NPs and villi. In the duodenum of RhB-treated mice, a deep overlapping between the villi and the RhB was observed at both 1 and 24 h after treatment. A higher magnified image confirms the penetration of the dye into the external side of the villi. Scale bar 50 µm.

Figure 5A, upper panels, shows representative images taken from gastric sections of mice sacrificed 1, 4, and 24 h after PLGA-PEG-RhB-NPs administration. Although a progressive reduction of the signal can be seen, it is important to underline that the anatomical localization of the signal remains almost exclusively confined outside the gastric cells that are characterized by the intense blue staining due to the presence of the nuclear dye Hoechst 33258. Opposite, the RhB alone, Figure 5A lower panels, deeply penetrated inside the gastric parenchyma as clearly evidenced by the purple staining due to the merge between the red and the blue signal. This is more pronounced at the first hour and, interestingly, it does not involve the whole structure of the stomach, but it is almost confined to the superficial region of the gastric mucosa (left part of the picture at 1 h). At the 4th hour, the signal is lower but more penetrated and homogeneously spread in the parenchyma, whereas after one day, as already demonstrated by ex vivo quantification (Figure 4B), the fluorescent intensity strongly decreased. The different pattern of staining shown at the 4th and 24th hour strongly suggests that, in spite of the gastric activity, PLGA-PEG-RhB-NPs remain stable enough to avoid the release of the free dye. This is in line with the results reported in Figure 1A by DLS in solutions mimicking gastric juice.

The stability of the nanoparticles inside the stomach is essential to transport any encapsulated drug to the intestine. In Figure 4B, the signal measurement along the whole small intestine was evaluated, whereas histological analysis was focused on the more proximal part of the intestine, the duodenal region. The duodenum is one of the most critical portions of the GI for the absorption of metabolites and drugs. The active uptake by mucosae villi and Peyer’s patches allows the absorption of many substances into the bloodstream and their consequent systemic distribution. Even if this process is required to provide energy and nutrients and to distribute therapeutic agents orally administered, it can be a hurdle for a localized gut delivery. In Figure 3, we have reported that the encapsulation in PLGA-PEG-RhB-NPs dramatically reduces the PTX absorption. This suggests that these kinds of NPs are able to pass through this first part of the intestine, maintaining their stability. Representative images from coronal sections of the duodenum from mice sacrificed 1 h after the treatment (lower panel on the left and higher magnification right in Figure 5B) shows that NPs are in the lumen and inside the intervilli space but are not absorbed by mucosa. A deeper interaction with villi can be seen at the 4th hour after treatment. However, even in this case, the red and blue signals are close but do not overlap. An overlapping was indeed clearly seen in distinction from mice treated with the same amount of RhB free at least up to the first 4 h after ingestion. A higher magnified picture furthermore confirms the restricted interaction between the red signal and the peripheral region of villi 1 h after treatment. As reported in the measurement of the intestinal levels by ex vivo scanning (Figure 4B), an almost complete disappearance of the red signal can be seen 24 h after the treatment in both experimental groups.

**Figure 6:**
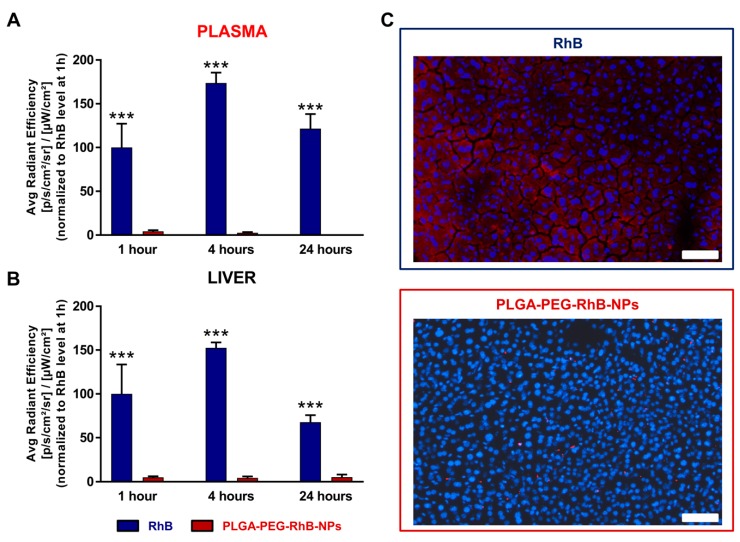
Signal measurement in (**A**) the plasma and (**B**) the liver of mice orally treated with RhB (blue bars) or PLGA-PEG-RhB-NPs (red bars). Animals were sacrificed at 1, 4, and 24 h after the treatment with the same dose of RhB. Five animals for each experimental group were used. In both graphics, quantification of the signal was normalized to the RhB level at 1 h and expressed as 100. The bars are the mean value ± SD (*n* = 5). The Student’s *t*-test was used to compare the levels between the two groups for each time point. *** *p* < 0.0001. (**C**) Representative images of the signal related to the dye in a section of liver from mice treated with PLGA-PEG-RhB-NPs (upper panel) or RhB alone (lower panel), both sacrificed 1 h after the treatment. Scale bar 50 µm.

By histology, we found that RhB-free treated mice showed deep red staining in villi, whereas the red signal remained in the lumen of the intestinal tube in animals receiving RhB with NPs. Since enterocytes in villi are tightly connected to the vessels, it is, therefore, possible to hypothesize that RhB can easily penetrate the circulatory tree. To confirm that NPs can dramatically reduce the passage from the small intestine to the bloodstream, we compared the RhB levels both in plasma and in the liver of mice treated with PLGA-PEG-RhB-NPs or RhB free. Figure 6A, where plasmatic levels of RhB-related signals were normalized to the value measured in mice treated with RhB-free during the first hour of analysis, clearly reveals that the nanoformulation (red bars) leads to an almost complete abolishment of hematic absorption of RhB. This striking difference between the two groups supports the hypothesis that these NPs are stable and almost completely eradicate the absorption of themselves and of the relevant cargo by gastric and intestinal mucosae. The fast and quite elevated half-life of RhB in the blood led to an expected accumulation of the dye in the main filter organ, the liver, Figure 6B. Similar to the results obtained from the blood, the animals treated with RhB exclusively showed a well detectable red signal in liver sections (Figure 6C).

**Figure 7:**
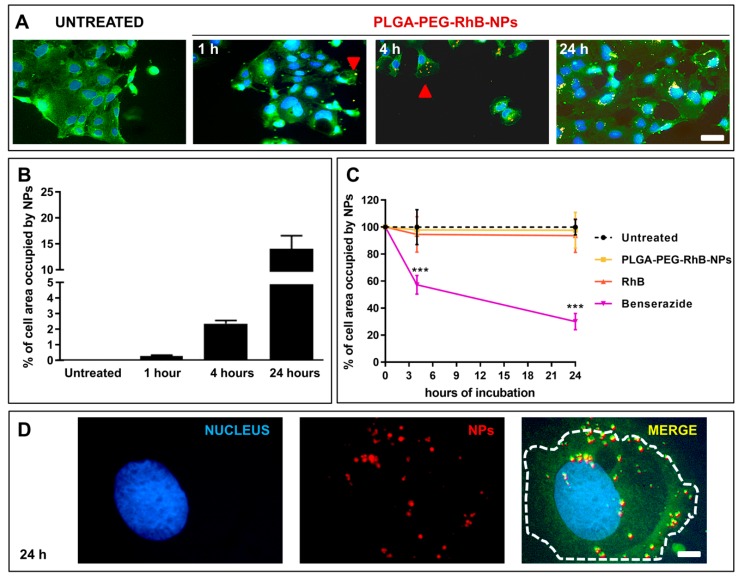
Longitudinal study to evaluate PLGA-PEG-RhB-NPs internalization in CaCo_2_ cells. (**A**) Low magnified pictures showing the progressive penetration of NPs in cells. Representative images have been selected from non-treated cells (NT, left panel). For each image, the nuclei were stained with Hoechst 33258 (blue), whereas high excitation with the laser at the wavelength of 488 nm will allow unveiling the border of the cells by exploiting their auto fluorescent profile (green). Starting from the 1st hour, it is possible to see orange spots obtained by the merge between the red signal referred to the RhB and the green background. These spots became more evident 4 and 24 h after incubation. Scale bar 70 µm. (**B**) Quantification of the percentage of the area (pixels) occupied by NPs for every single cell at the different time-points. Data are expressed as mean value ± SD, *n* = 10. (**C**) Quantification of the viability of the cells of CaCo_2_ after exposure to RhB (orange line), PLGA-PEG-RhB-NPs (yellow line), and Benserazide as inner control (purple line) measured by RealTime-Glo™ MT Cell Viability Assay (Promega kit) 4 and 24 h after incubation The values obtained from non-treated cells were normalized to 100 for each time point. Values are expressed as mean ± SD (*n* = 6). The Student’s *t*-test was used to compare the levels among the two groups for each time point. *** *p* < 0.0001 compared to NT. (**D**) Higher magnification pictures showing the same field of view achieved 24 h after PLGA-PEG-RhB-NPs incubation. In the left panel, the cell nucleus, in the middle panel, the red spots associated with NPs, and in the right panel, the merge between the three channels. The dotted line is the border of the cell. Scale bar 15 µm.

The last part of the study was carried out to investigate if these NPs were able to penetrate into CaCo_2_ cells and where they localize inside the cells. This experiment was aimed at exploring the possible application of our results to future local treatment of colorectal cancer. Figure 7A shows the progressive process of internalization of NPs (orange spots). The quantification of the occupied area of NPs inside the cell cytoplasm is reported in Figure 7B. Progressive penetration of NPs occurs and, at the 4th hour after incubation, they already occupied the 2% to 3% of the whole cell area. Although relatively low, this percentage in terms of a potential release of a therapeutic cargo cannot be considered negligible. The red arrow in Figure 7A and the higher magnified picture in Figure 7D confirm that NPs are deeply penetrated (orange spots) and the deep internalization of NPs inside the cell cytoplasm starting from the 4th hour of incubation. Moreover, the cell viability assay confirmed the safety of the materials we selected for this study. Indeed, neither RhB nor PLGA-PEG-RhB-NPs alone modify the healthiness of the cells for the whole duration of the treatment (Figure 7C).

## 4. Conclusions

The current study sought to evaluate the effect of the nanocarrier on the transport of a drug and its behavior within the gastrointestinal tract and absorption to the bloodstream. It is important to note, however, that although RhB was originally used as a tracer to visualize NPs. The results obtained by comparing the biodistribution in mice of the free fluorophore and linked to the NPs is of further relevance for future developments. It is, in fact, interesting how PLGA-PEG-NPs manage to preserve the gastroduodenal absorption by using chemically different molecules along with different loading approaches to the nanocarrier. The low systemic exposure and, at the same time, equivalent drug concentration at the intestinal level, with even a trend to increase in the colon 4 h after the treatment, could have a significant positive outcome on the safety of a wide range of drugs targeting inflammatory and neoplastic diseases.

## Figures and Tables

**Table 1:** NPs characterization and process yield. Data represent mean ± SD (*n* = 3).

Formulations	Size (nm ± SD)	PDI	Z-Potential (mV ± SD)	Process Yield (%, *w*/*w* ± SD)
PLGA-PEG-NPs	190.3 ± 12.7	0.078 ± 0.032	+17.9 ± 5.9	53.3 ± 7.7
PLGA-PEG-RhB-NPs	205.6 ± 18.6	0.168 ± 0.030	−18.2 ± 1.5	50.1 ± 13.4
PTX-PLGA-PEG-RhB-NPs	201.6 ± 26.2	0.205 ± 0.032	−13.3 ± 1.7	39.3 ± 7.3

## Supplementary Materials

The following are available online at https://www.mdpi.com/1999-4923/11/12/658/s1, Figure S1: STEM image of PTX-PLGA-PEG-RhB-NPs, Figure S2: Release kinetics of PTX loaded NPs in PBS containing 0.2 *v*/*v*% Tween 80. PTX-PLGA-PEG-RhB-NPs equivalent to 8.75 μg of PTX were suspended in 1 mL of release medium and incubated at 37 °C with constant agitation. At predetermined time points (0.5, 1, 2, 4, 6 and 24 h), the NPs were separated by centrifugation and the supernatant analyzed by HPLC. The results are the mean value ± SD (*n* = 4).
