# Supplementary material for: Monitoring the Fate of Orally Administered PLGA Nanoformulation for Local Delivery of Therapeutic Drugs
Source: Pharmaceutics. 2019 Dec 6;11(12):658. doi: 10.3390/pharmaceutics11120658 (PMC6955864; doi:10.3390/pharmaceutics11120658)
Supplement: Supplementary file 1 [file pharmaceutics-11-00658-s001.pdf]

pharmaceutics

Supplementary Material: Monitoring the Fate of Orally Administered PLGA Nanoformulation for Local Delivery of Therapeutic Drugs

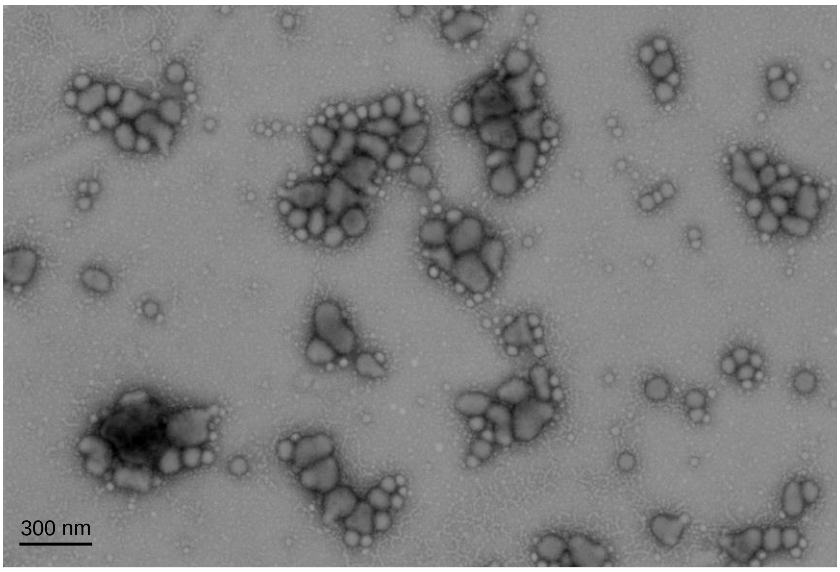

Figure S1. TEM image of PLGA-PEG-RhB-PTX nanoparticles.

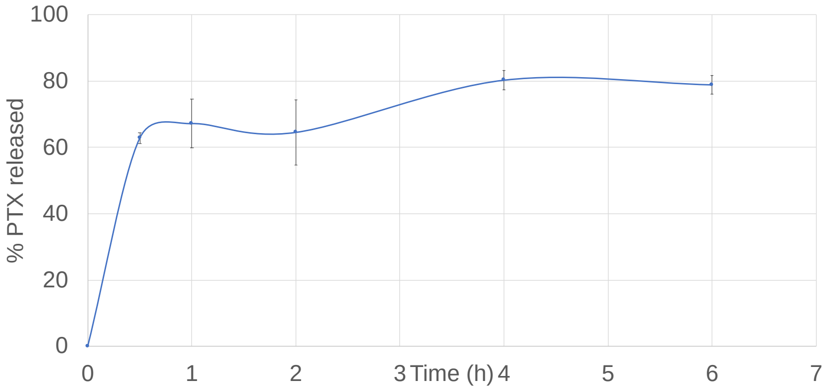

Figure S2. Release kinetics of PTX from NPs in xx media. PLGA-PEG-RhB-PTX nanoparticles equivalent to xx µg of PTX were suspended in x mL of medium and incubated at 37 °C with constant agitation. At predetermined time points, the suspension was centrifuged to separate NP pellets and supernatants. Then 0.8 mL of supernatant was sampled and replaced with the same volume of fresh medium. The NP pellet was resuspended and returned for further incubation. The sampled supernatant was analyzed as sampled (PBS and Tween/PBS), with the addition of an equal volume of acetonitrile (PBS-AcN treated), or after extraction with ethyl acetate (FBS/PBS).
